# Tryptophan Catabolism and Response to Therapy in Locally Advanced Rectal Cancer (LARC) Patients

**DOI:** 10.3389/fonc.2020.583228

**Published:** 2020-10-15

**Authors:** Sara Crotti, Alessandra Fraccaro, Chiara Bedin, Antonella Bertazzo, Valerio Di Marco, Salvatore Pucciarelli, Marco Agostini

**Affiliations:** ^1^Nano-Inspired Biomedicine Laboratory, Institute of Paediatric Research—Città della Speranza, Padua, Italy; ^2^Department of Chemical Sciences, University of Padua, Padua, Italy; ^3^Department of Pharmaceutical and Pharmacological Sciences, University of Padua, Padua, Italy; ^4^First Surgical Clinic Section, Department of Surgical, Oncological and Gastroenterological Sciences, University of Padua, Padua, Italy

**Keywords:** tryptophan, rectal cancer, IDO1, TDO2, TPH1, kynurenine pathway, serotonin pathway

## Abstract

In locally advanced rectal cancer patients (LARC), preoperative chemoradiation improves local control and sphincter preservation. The response rate to treatment varies substantially between 20 and 30%, and it is an important prognostic factor. Indeed, nonresponsive patients are subjected to higher rates of local and distant metastases, and worse survival compared to patients with complete response. In the search of predictive biomarkers for response prediction to therapy in LARC patients, we found increased plasma tryptophan levels in nonresponsive patients. On the basis of plasma levels of 5-hydroxy-tryptophan and kynurenine, the activities of tryptophan 5-hydroxylase 1 (TPH1) and indoleamine-2,3-dioxygenases 1 (IDO1)/tryptophan-2,3-dioxygenase (TDO2) have been obtained and data have been correlated with gene expression profiles. We demonstrated that TDO2 overexpression in nonresponsive patients correlates with kynurenine plasma levels. Finally, through the gene expression and targeted metabolomic analysis in paired healthy mucosa-rectal cancer tumor samples, we evaluated the impact of tryptophan catabolism at tissue level in responsive and nonresponsive patients.

## Introduction

Cancer is a major cause of death in industrialized countries, and colorectal cancer (CRC) is one of the most common tumors in both male and female (1). About 30% of CRC cases concern the rectal tract of the large intestine, approximately the last 15 cm of the intestinal tract. In the most advanced stages of the disease, the tumor delocalizes and begins its proliferation in areas of the body different from the one in which it arose. Due to the different blood and lymph node ducts to which they are connected, colon cancer mainly develops liver metastases while rectal cancer develops, in addition to the liver ones, also thoracic metastases. Prevention and diagnosis strategies for rectal and colon cancer are mostly the same; the planned therapy, however, is shared only for some traits.

Preoperative chemoradiotherapy (pCRT) is worldwide accepted as a standard treatment for locally advanced rectal cancer (LARC) with stage II and III aiming at improving local tumor control, and inducing tumor downsizing and downstaging (2). Standard treatment includes administration of ionizing radiation for 45–50.4 Gy associated with 5-fluorouracil administration during radiation therapy and few modifications (e.g., adding Oxaliplatin) are introduced to ameliorate primary tumor response and, consequently, patients' outcome. Typically, complete pathologic response rate to pCRT is between 20 and 30% (3). It follows that patients with *a priori* resistant tumor should not be included in the treatment, which is associated with substantial adverse effects and higher rates of local and distant metastases. The search of predictive biomarkers for response prediction to therapy in rectal cancer would improve the patients' management. In this frame, in adjunction to clinical features (4, 5), the potentiality of liquid biopsy has been extensively employed to identify circulating predictive biomarkers (6, 7). Indeed, a number of putative biomarkers including proteins (8, 9), circulating peptides (10), and circulating tumor cells or nucleic acids (11–13) have been proposed. However, recently an increased focus on the tumor microenvironment offered further opportunities to understand the tumor response biology and the relations between pCRT and the radiation-induced response (14, 15), together with tumor-specific immune response (16).

**Figure 1:**
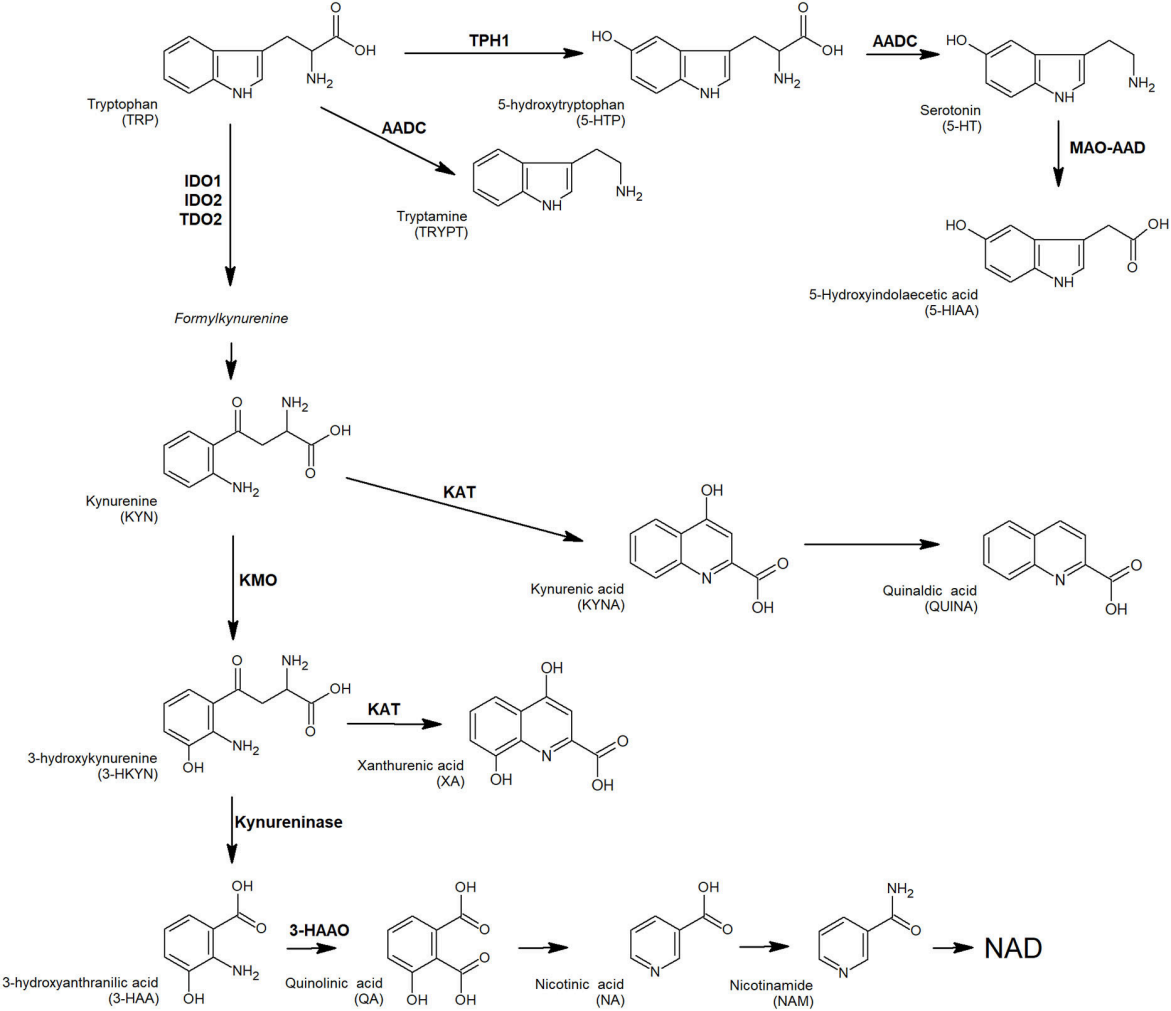
TRP and its key catabolites (TRYCATs) produced via three biochemical pathways: the kynurenine pathway, tryptamine pathway, and serotonin pathway. Enzymes involved: AADC, aromatic L-amino acid decarboxylase; AD, aldehyde dehydrogenase; 3-HAAO, 3-hydroxyanthranilate 3,4-dioxygenase; IDO1, indoleamine-2,3-dioxygenases 1; IDO2, indoleamine-2,3-dioxygenases 2; KAT, L-kynurenine aminotransferase; KMO, kynurenine monooxygenase; MAO-A, monoamine oxidase A; TDO, tryptophan-2,3-dioxygenase; TPH1, tryptophan hydroxylase 1.

Physiologically, several pro-inflammatory mediators and T cells cytotoxic activity are modulated by tryptophan (TRP) and its metabolites, as an adaptation mechanism to restrict excessive acute immune response in tissues (17). Tryptophan is the precursor of several active compounds, collectively named TRYCATs (Figure 1). At the tumor microenvironment, TRP catabolism is promoted by indoleamine 2,3-dioxygenase (IDO1) overexpression under pro-inflammatory conditions, and it plays an important role in modulating antitumor immune response (18–20). In CRC, IDO1 expression at the tumor invasion front correlates with disease progression and worse clinical outcome (21) and is associated with the frequency of liver metastases (22). As observed in other tumors, local TRP depletion in CRC plays an important role in either antitumor immune response suppression or cancer cell proliferation/survival support (23–25). Beside IDO1, a second enzyme involved in TRP metabolism, tryptophan-2,3-dioxygenase (TDO2) is expressed in a significant proportion of human tumors and is involved in proliferation, migration, invasion, and immunoresistance (26–28). A valuable way to measure local catabolism is represented by plasma or blood quantification of TRP and its main metabolites, i.e., 5-hydroxy-tryptophan (5-HTP), kynurenine (KYN), and serotonin (5-HT). Consequently, circulating levels of TRP can be used as a “proxy” for tumor microenvironment metabolism.

In this frame, we highlighted that CRC-associated inflammation is capable of modulating circulating levels of TRP and its metabolites along the adenoma–carcinoma sequence. Indeed, decreased TRP concentration and increased IDO1 and tryptophan hydroxylase 1 (TPH1) enzymatic activities were detectable in plasma samples concomitant to precancerous lesion (high grade-adenomas) or in association to risk factors (inflammatory bowel diseases) (29). Moreover, we defined the TRP catabolism as a possible source of prognostic marker for familial adenomatous polyposis patients, based on IDO1 and TPH1 activity with high sensitivities and specificities (up to 92%) (30). Agostini et al. have firstly suggested the link between IDO1 and chemoresistance of rectal cancer patients in a *de novo* meta-analysis on rectal tumor tissues (31). IDO1 and other two genes involved in the immune system pathway (AKR1C3 and CXCL10) have been identified as a gene set associated with pCRT response and survival. Consistently with these results, we focused our attention on TRP metabolism as a hallmark of immune host response modulation in LARC. In this study, both at circulating and at tissue level, we investigated metabolic and genetic markers of the TRP catabolism before pCRT in LARC patients in order to find out new predictive biomarkers measuring the response to therapy.

## Materials and Methods

### Chemicals

Isotopically labeled internal standards d_5_-tryptophan (TRPd, 98.8%), d_4_-serotonin (5HTd, 98.7%), and d_5_-kynurenic acid (KINAd, 99.2%) were purchased from CDN isotopes (Quebec, Canada) while ^13^C_6_-nicotinamide (NAmC, 99.4%) was purchased from Sigma Aldrich (Milan, Italy). Analytical standards for tryptophan (TRP), kynurenine (KYN), 5-hydroxy-tryptophan (5-HTP), serotonin (5-HT), tryptamine (Tryp), kynurenic acid (KYNA), quinaldic acid (QA), xanthurenic acid (XA), 3-hydroxyanthranilic acid (3-HAA), 5-hydroxyindoleacetic acid (5-HIAA), nicotinic acid (NA), quinolinic acid (QuiA), and nicotinamide (NAm) have been purchased from Sigma Aldrich (Milan, Italy). LC-MS-grade solvents (acetonitrile, methanol, chloroform, isopropanol), and suprapure trifluoroacetic acid (TFA) were purchased from Romil. TRIzol™ reagent was obtained from Thermo Fisher Scientific.

### LC-MS/MS and LC-UV/FLD Analyses

Mass spectrometry measurements were performed by using an API 4000 triple quadrupole mass spectrometer (AB SCIEX, MA, USA) coupled to an Ultimate 3000 UPLC system (Thermo Fisher). Analyzed metabolites were TRP, KYN, 5-HTP, 5-HT, Tryp, KYNA, QA, XA, 3-HAA, 5-HIAA, NA, QuiA, and NAm. Scheduled MRM transitions, instrumental parameters, and chromatographic conditions were reported in Supplementary Material S1.

HPLC-UV/FLD analyses of plasma samples to detect and quantify TRP, KYN, 5-HTP, and 5-HT were performed as previously reported (29).

### Sample Collection

This study was conducted according to the principles expressed in the Declaration of Helsinki. Biological specimens (de-personalized plasma and tissue biopsies) were obtained from the Tissue Biobank of the First Surgical Clinic of Padua Hospital (Italy). The protocol was approved by the ethics committee of the institution (Comitato Etico del Centro Oncologico Regionale, Approved Protocol Number: P448). Selected samples were obtained from rectal cancer patients before preoperative chemoradiotherapy (pCRT) which consisted of external-beam radiotherapy (>6 MV) using a conventional fractionation (>50 Gy in 28 fractions, 1.8 Gy per day, 5 sessions per week) and 5-fluorouracil administered as neoadjuvant chemotherapy drug by bolus or continuous venous infusion. Elective surgery was performed after 7 weeks (median value) to completion of preoperative chemoradiotherapy (interquartile range 6–8 weeks), and patients' response to pCRT was evaluated after histological evaluation of surgical resection as the tumor regression grade (TRG) according to Mandard et al. (32). All demographic and clinical data are presented in Table 1.

**Table 1:** Clinical and demographic characteristics of all LARC patients.

**Characteristic**		***N***	**%**
Age	Median (range yrs.)	66 (31–79)	
Sex	Male	52	63%
	Female	30	37%
Tumor distance from anal verge	≤ 7 cm	26	32%
	>7 cm	39	48%
	Not available	17	20%
TRG	1–2	37	45%
	3–5	45	55%
Specimens	Plasma	45	
	Tissues	69	
Paired samples	Plasma–tumor samples	32	
	Healthy mucosa–tumor samples	13	

*Tumor regression grade (TRG) is calculated according to Mandard et al. (32). TRG 1–2, responders; TRG 3–5, nonresponders*.

### Sample Preparation

Healthy rectum mucosa and tumor pre-therapy biopsies were processed to isolate total RNA by TRIzol™ reagent following the manufacturer's protocol. After chloroform addition, the aqueous upper layer was transferred for the subsequent gene expression analysis while the lower organic phase containing the interphase layer was stored at −20°C for metabolite quantification. RNA concentration and purity were estimated as the ratio 260/280 nm by the NanoDrop 2000 spectrophotometer (Thermo Scientific, USA). Only samples with a ratio between 1.7 and 2.1 were considered suitable for downstream analysis. Reverse transcription and quantitative real-time PCR (qPCR) were performed as described in Supplementary Material S2.

TRP and its metabolites were extracted from the lower organic phase obtained during the RNA isolation by adding an equal volume of acidified cold water (0.05% TFA) containing known amounts of the following internal standards: TRPd, 5HTd, KINAd, and NAmC. The mixture was centrifuged at 4°C for 5 min (12,000 rpm) using a Heraeus Fresco 21 centrifuge (Thermo Electron Corp.). Extracted analytes were transferred into a new tube and dried under vacuum. The residual organic layer, containing the interphase, was treated with ethanol (300 μL) to eliminate DNA, and residual protein pellet was extracted according to TRIzol™ manufacturer's instructions. Total protein amount was finally quantified by the Pierce BCA Protein Assay Kit (Thermo Fisher).

### Statistical Analysis

Statistical analysis was performed with GraphPad Prism, version 5.00, 2007 (La Jolla, CA, USA). Normality of data was evaluated using the D'Agostino-Pearson omnibus normality test, and parametric (or nonparametric) statistical analyses were completed accordingly. Spearman rank test was used to determine the strength and direction of the relationship between variables.

## Results and Discussion

### Circulating TRP Metabolite Levels and Response to Therapy

**Figure 2:**
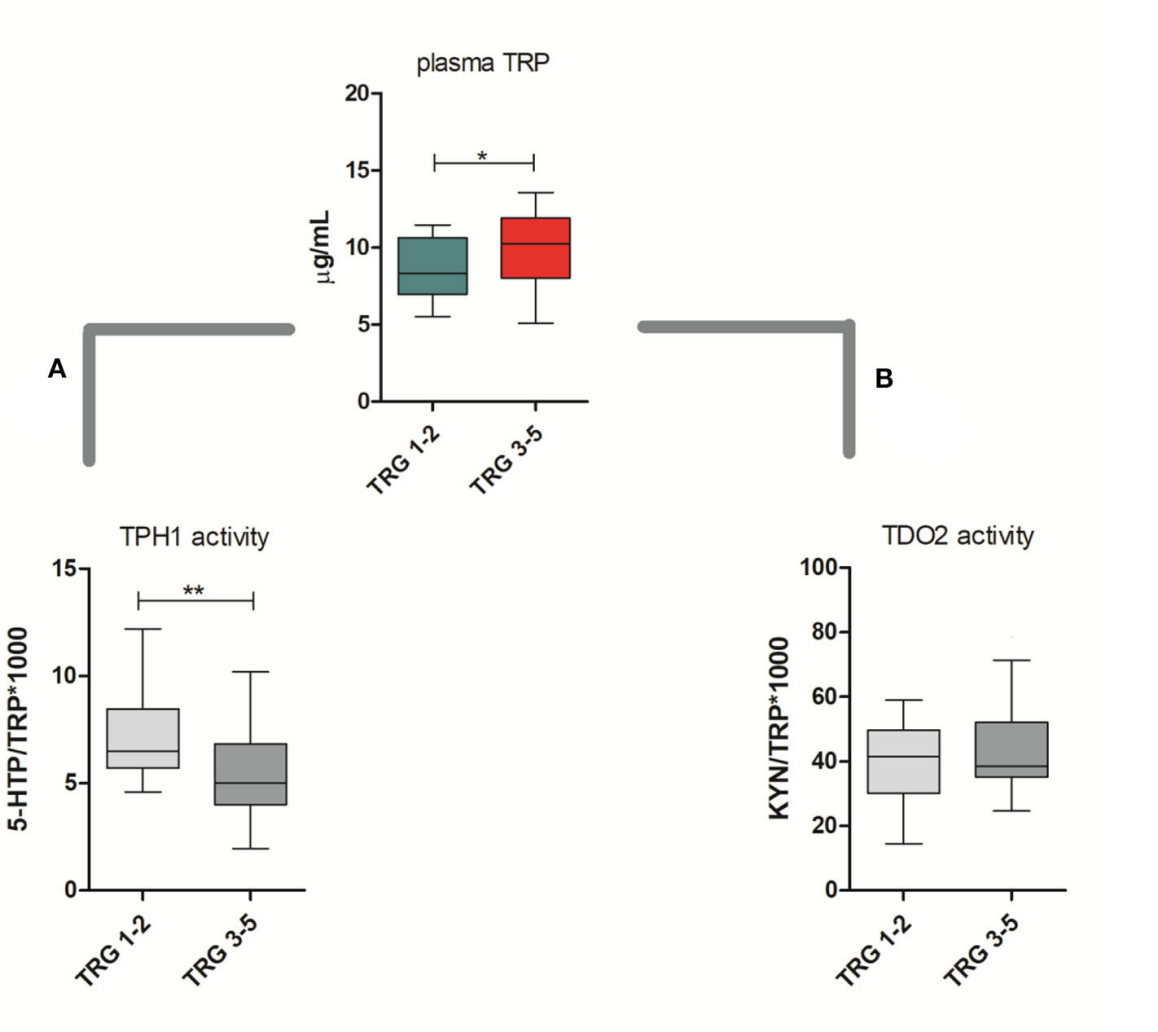
Circulating plasma levels of tryptophan (TRP) in responsive (TRG 1–2) and nonresponsive (TRG 3–5) rectal cancer patients. Calculated TPH1 activity **(A)** and IDO1/TDO2 **(B)** activities are reported. Mann–Whitney, *= *p* < 0.05, ** = *p* < 0.01.

TRP catabolism in 45 LARC patients was assessed through plasma level quantification of TRP and its major metabolites (KYN, 5-HTP, and 5-HT) by means of HPLC-UV-VIS/FLD analysis. Usually, TRP plasma levels are physiologically regulated by the hepatic TDO2 enzyme. However, under non-physiological conditions (e.g., in presence of inflammation or cancer), overexpression of IDO1/TDO2 enzymes can actively contribute to TRP catabolism. The median TRP concentration was 9.01 μg/mL (8.68–10.01, 95% CI) which is—as expected—very close to the TRP concentration we observed in our previous investigation for control (i.e., healthy subjects) plasma samples (29). Indeed, we already demonstrated that TRP catabolism increases more in people affected by colon cancer than those affected by rectal cancer. Differently, in the present study, the cohort of rectal cancer patients was collected to check for differences between TRG 1–2 and TRG 3–5 patients and not for diagnostic evaluation (i.e., comparison with healthy subjects). Data reported in Table 2 suggest that a statistically significant increase of TRP in TRG 3–5 patients is present, together with an increasing trend of KYN levels. No difference is present between 5-HTP and 5-HT plasma levels. When IDO1/TDO2 and TPH1 enzymatic activities are estimated from these data, following the usual approach (29), a lower TPH1 enzymatic activity (*p* < 0.01) resulted for TRG 3–5 patients (Figure 2A, box-plots). This decrease underlines a possible involvement of serotonin pathway in tumor response, while the IDO1/TDO2 activity, which is an estimation of kynurenine pathway, shows only a nonstatistically significant increasing trend (Figure 2B, box-plots).

**Table 2:** Tryptophan (TRP), kynurenine (KYN), 5-hydroxy-tryptophan (5-HTP), serotonin (5-HT) plasma levels and tumor tissue expression of enzymes involved in TRP metabolism.

**Plasma metabolite levels**					
	**TRG 1–2 (*****n*** **=** **17)**		**TRG 3–5 (*****n*** **=** **28)**		***p*-value**
	**Median**	**Q1, Q3**	**Median**	**Q1, Q3**	
TRP μg/mL	8.43	7.06, 10.71	10.24	8.37, 11.93	<0.05
KYN μg/mL	0.31	0.24, 0.46	0.41	0.30, 0.53	
5HTP μg/mL	0.06	0.05, 0.07	0.05	0.04, 0.06	
5HT μg/mL	0.01	0.005,0.01	0.01	0.001, 0.02	
**Gene expression of involved enzymes**					
	**TRG 1–2 (*****n*** **=** **25)**		**TRG 3–5 (*****n*** **=** **27)**		***p*****-value**
	**Median**	**Q1, Q3**	**Median**	**Q1, Q3**	
IDO1 (RQ)	66.87	23.08, 138.6	57.46	25.73, 98.07	
TDO2 (RQ)	67.21	40.12, 148.7	185.8	57.74, 250.0	<0.05
TPH1 (RQ)	1.68	0.66, 6.44	2.97	0.82, 12.0	

*RQ, relative quantitation*.

To evaluate this hypothesis, we decided to perform a gene expression analysis of IDO1, TDO2, and TPH1 in order to clarify whether in TRG 3–5 patients the decrease in TPH1 systemic activity actually means a downregulation of TPH1 gene expression. However, as underlined by gene expression data reported in Table 2, there is no difference for TPH1 and IDO1 between TRG 1–2 and TRG 3–5 patients. On the contrary, TDO2 shows instead a statistically significant increase in TRG 3–5 patients (*p* < 0.05). Even after data normalization to healthy rectal mucosa (*n* = 17 samples, not paired), TDO2 was still overexpressed in TRG 3–5 patients only (tumor/healthy mucosa average ratio: 1.035 for TRG 1–2 and 2.230 for TRG 3–5), while TPH1 was equally downregulated in both patients' groups (tumor/healthy mucosa average ratio: 0.015 for TRG 1–2 and 0.022 for TRG 3–5). To further verify whether these metabolic and gene expression alterations may be consistent, Spearman's rank correlation test was employed to analyze the results from only the paired plasma-tumor samples (*n* = 32). Plasma levels of detected metabolites (both precursors TRP and its products KYN, 5-HTP, and 5-HT) and the calculated enzymatic activities of IDO1, TDO2, and TPH1 have been compared against their quantified genes expression. Obtained results indicated that in rectal cancer patients, KYN plasma levels are strongly correlated with TDO2 gene expression (*r* = 0.6026, *p* < 0.001, *n* = 32) and, after patients' stratification according to their response to therapy, only those having TRG 3–5 still showed a positive correlation between KYN and TDO2 (*r* = 0.556, *p* < 0.05, *n* = 17).

Following the same procedure, we found that neither IDO1 nor TPH1 gene expressions were correlated with their metabolites or calculated activities in rectal cancer patients. Other authors previously showed this discrepancy for IDO1 (33). To explain this behavior, it should be noted that that enzymatic process coordination depends upon temporal regulation of both substrates and enzymes. Moreover, changes in mRNA levels of IDO1 and TPH1 just indicate cell metabolic changes and may moderately correlate with changes in enzymes activity. Consequently, as for most of proteins, disparity between mRNA levels and protein abundance make it difficult to predict real activity for these enzymes (34).

### Local Quantification of TRP Metabolites Better Reflects Gene Expression

To clarify whether it is possible to correlate the amount of TRP and its metabolites to enzyme activity and their gene expression in LARC patients, we developed and validated an analytical method for the simultaneous evaluation of TRP catabolism at both metabolic and genetic levels. By this method, metabolite quantification and gene expression analysis were obtained in the same tissue samples, by means of a sequential extraction procedure. In brief, paired biopsies (healthy mucosa and rectal cancer counterpart) have been extracted with TRIzol™ following the manufacturers' protocol. The resultant upper aqueous phase was used for quantitative real-time PCR of IDO1, TDO2, and TPH1 genes, while the lower organic phase was added by the four internal standards (TRPd, 5HTd, KINAd, and NAmC) before metabolite extraction (see Sample Preparation section for details).

A total of 13 metabolites have been quantified by a single scheduled LC-MRM analysis. These metabolites were tryptophan (TRP), kynurenine (KYN), 5-hydroxy-tryptophan (5-HTP), serotonin (5-HT), tryptamine (Tryp), kynurenic acid (KYNA), quinaldic acid (QA), xanthurenic acid (XA), 3-hydroxyanthranilic acid (3-HAA), 5-hydroxyindoleacetic acid (5-HIAA), nicotinic acid (NA), quinolinic acid (QuiA), and nicotinamide (NAm). Instrumental parameters and scheduled transitions employed to quantify and qualify metabolites are resumed in Supplementary Material S3. Good performances in terms of LLOQ, LOD, CV %, and accuracy % for all metabolites have been obtained, with exception to Tryp and 3-HAA (Supplementary Material S3). For these two metabolites, the present method was not able to provide enough reproducibility and then quantitative results should be considered as approximate.

**Figure 3:**
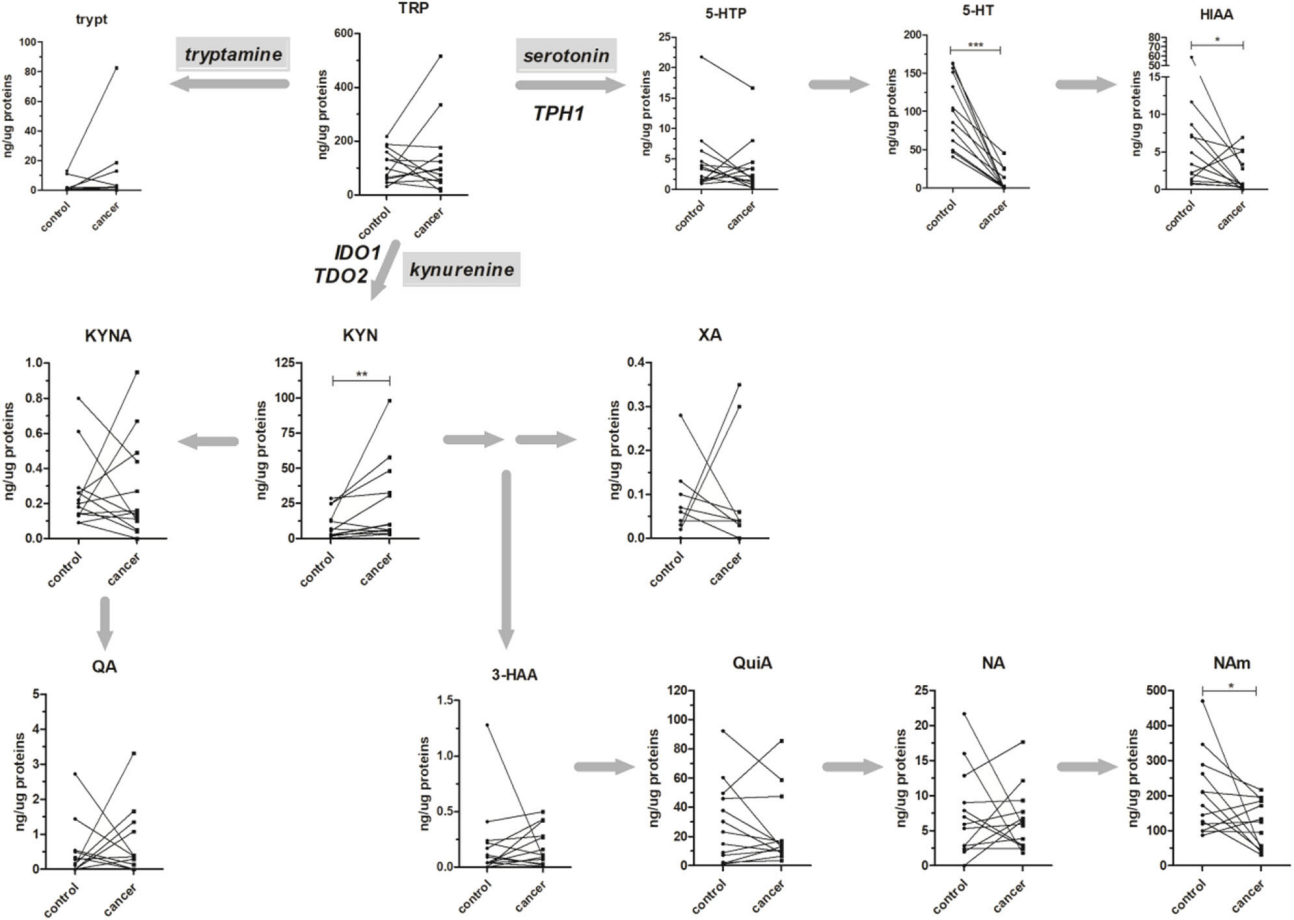
Absolute amounts (presented as ng of metabolite per μg of total proteins) of TRP along the kynurenine and serotonin pathways in 13 healthy mucosa (control) and rectal cancer (cancer) paired samples. The tryptamine metabolic pathway is presented for completeness. Matched samples are connected together. Results from Wilcoxon signed rank test are reported (*p < 0.05; **p < 0.01; ***p < 0.001).

The method was applied to the analysis of 13 paired healthy mucosa/rectal cancer samples, and obtained results are reported in Figure 3. All the major metabolites along the kynurenine and serotonin pathways have been quantified; in addition, Tryp has been included in the present study for completeness, even if tryptamine pathway accounts only for the <1% of TRP catabolism (35). For both types of samples, no difference was present in the TRP tissue level (average values: 97.98 and 95.06 ng/μg of proteins, for healthy mucosa and rectal cancer, respectively). However, in cancer tissues a trend of decrease along the serotonin pathway was observable for 5-HTP and further confirmed by the statistically significant decrease of 5-HT (*p* < 0.001, Wilcoxon signed-rank test) and its final catabolite 5-HIAA (*p* < 0.05, Wilcoxon signed-rank test). This strong decrease in serotonin level is reasonably due to the lack of enterochromaffin cells, which are normally present in healthy rectal mucosa but not in cancer tissue. These cells are a specialized subset of enteroendocrine cells and the largest producer of 5-HT in the body (~95%), which is critical to gastrointestinal motility (36). On the contrary, KYN increased significantly in rectal cancer tissues (*p* < 0.01, Wilcoxon signed-rank test). This increase could be the result of a diminished TRP consumption along the serotonin pathway and could explain, at least theoretically, the unaltered total TRP levels in rectal cancer tissues. Increased KYN levels in colon cancer tissues and human colon cancer cells have been recently correlated with the tumor proliferation through the activation of the aryl hydrocarbon receptor (AHR) (37). KYN exerts also immuno-modulating effects at the tumor microenvironment (38) and is the precursor of quinaldic acid (QA), xanthurenic acid (XA), and nicotinamide (NAm) (Figure 3). QA and XA levels were comparable between healthy mucosa and rectal cancer samples. On the contrary, NAm, which is the precursor for redox cofactor NAD^+^, was decreased in rectal cancer samples (*p* < 0.05, Wilcoxon signed-rank test). NAm decrease may be a hallmark of increased energetic demand in tumor; indeed, cancer cells are able to reprogram their metabolism (nutrient uptake, intracellular metabolism, and gene expression) for sustaining survival, growth, and metastasis. In particular, to satisfy their NAD^+^ demand, tumors can overcome the limitation of a *de novo* synthesis from TRP and adopt a salvage pathway, which “recycles” existing NA and NAm (39).

The gene expression of rate-limiting step enzymes IDO1, TDO2, and TPH1 and their calculated activity were finally obtained for each sample (Table 3). For all the three enzymes, the mean of normalized RQ (cancer/healthy mucosa) was calculated and presented as fold change (F.C.). Obtained data revealed that in rectal cancer samples the kynurenine pathway was characterized by TDO2 overexpression, while IDO1 expression remained practically unchanged (using a 1.5-fold ratio criterion). These data are consistent with others reported in literature, in which TDO2 expression in several human tumor cell lines and tissues has been demonstrated (26, 28, 40–42). Similarly, a rise in the calculated enzymatic activity (KYN/TRP^*^1,000) in rectal cancer tissues compared to mucosa was detected (mean activity: 111 and 230 for healthy mucosa and rectal cancer, respectively; *p* < 0.05, Wilcoxon signed-rank test). This increase positively correlated with KYN produced and negatively correlated with the enzyme substrate TRP (Table 3) suggesting that, in rectal cancer, TDO2 may be responsible for increased KYN levels.

**Table 3:** Normalized (tumor/healthy mucosa) gene expression (*nRQ*) for all paired samples.

**All samples (*****n*** **=** **13 pairs)**					
	**Mean**	**Min, Max**	**Trend in tumor**	**Correlation with metabolites**	***p*-value**
IDO1 (nRQ)	0.71	0.10, 2.41	=	NAm (*r* = 0.424)	<0.05
TDO2 (nRQ)	1.56	0.09, 10.04	↑		
Calculated activity	230 (111)	8.6, 636 (2.19, 441)	↑	KYN (*r* = 0.803) TRP (*r* = −0.461)	<0.0001 <0.05
TPH1 (nRQ)	0.43	0.001, 3.65	↓	5-HT (*r* = 0.810)	<0.0001
Calculated activity	36.3 (40.7)	1.2, 148 (8.6, 115.7)	=	5-HTP (*r* = 0.680)	<0.0001

*Data are presented as mean fold change and minimum and maximum (Min, Max) values. Enzymatic activity is reported as calculated mean value for rectal cancer samples (healthy mucosa) and relative minimum, maximum. “Trend in tumor” column highlights fold changes at least >1.5 (or <0.67). Correlation between gene expression and TRP metabolites was evaluated using Spearman's rank test and only significant results are reported*.

Most importantly, the THP1 downregulation observed for this group of rectal cancer is consistent with that previously observed in the plasma–tissue correlation (actual fold change: 0.43). Differently to the previous observation, however, actual 5-HT tissue levels strongly correlated with THP1 expression and the calculated THP1 activity positively correlated with 5-HTP production (Table 3). Collectively, these results indicated that local quantification of TRP metabolites better reflects gene expression and enzymatic activity.

### Preliminary Correlation Between TRP Metabolism and Response Prediction

In our first analysis on paired plasma–tissue samples, we suggested that a decreased THP1 activity together with TDO2 overexpression was a common hallmark of lack of response to therapy in rectal cancer patients (Figure 2 and Table 2). In our second analysis on 13 paired control/cancer samples, we aimed at verifying these results and at correlating them to metabolite production at the tissue level. Again, we found that, after stratification of gene expression data, IDO1 was substantially unchanged in TRG 1–2 and TRG 3–5 patients, while TDO2 overexpression was peculiar of nonresponsive patients only (Table 4). Consistently to TDO2 overexpression, an increase in the calculated enzymatic activity along the kynurenine pathway has been detected in these patients (136 vs. 311, for TRG 1–2 and TRG 3–5, respectively). TPH1-normalized gene expression decreased in both TRG 1–2 patients (nRQ = 0.19) and TRG 3–5 patients (nRQ = 0.66), even if in the latter the decrease was less consistent. Conversely, calculated THP1 activity showed an opposite trend (43 vs. 29, for TRG 1–2 and TRG 3–5, respectively).

**Table 4:** Normalized (tumor/healthy mucosa) gene expression (*nRQ*) after patients' stratification according to their TRG (responders: TRG 1–2; nonresponders: TRG 3–5).

	**TRG 1–2 (*****n*** **=** **6 pairs)**		**TRG 3–5 (*****n*** **=** **7 pairs)**		
	**Mean**	**Min, max**	**Mean**	**Min, max**	**Trend in TRG 3–5**
IDO1 (nRQ)	0.66	0.13, 2.00	0.789	0.10, 2.41	=
TDO2 (nRQ)	0.88	0.40, 1.99	2.13	0.09, 10.04	↑
Calculated activity	136	8.6, 292.5	311	11.6, 636.6	↑
TPH1 (nRQ)	0.19	0.002, 0.44	0.66	0.001, 3.65	↑
Calculated activity	43	1.2, 149	29	2.6, 94.7	=

*Data are presented as mean fold change and minimum and maximum (Min, Max) values. “Trend in tumor” column highlights fold changes at least >1.5 (or <0.67). Enzymatic activity is reported as calculated mean value for rectal cancer samples (healthy mucosa) and relative minimum and maximum*.

**Figure 4:**
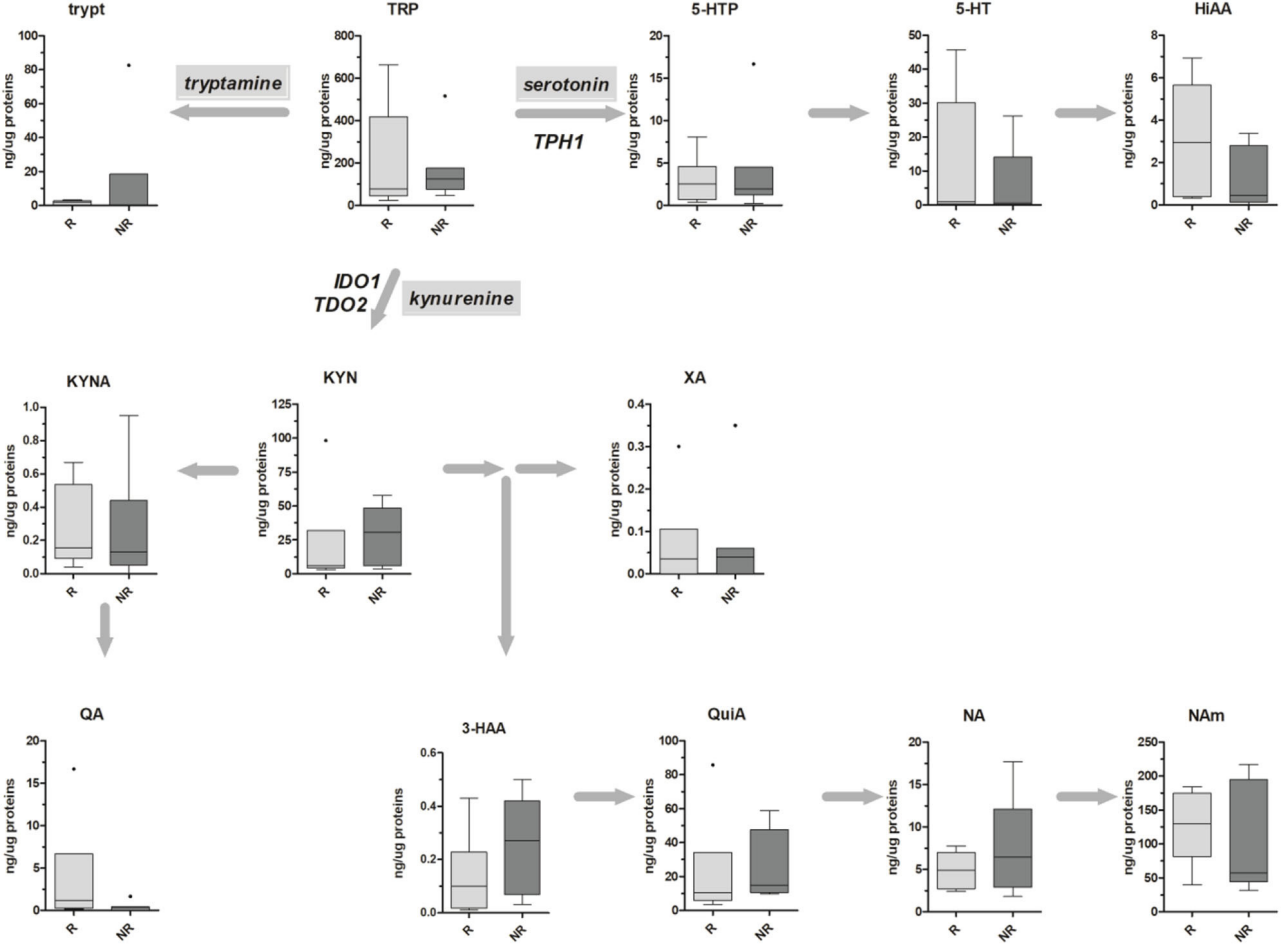
Box-plots representing the absolute amounts (presented as ng of metabolite per μg of total proteins) of TRP along the kynurenine and serotonin pathways in six TRG 1–2 (responders, R) and seven TRG 3–5 (nonresponders, NR) rectal cancer patients. Outliers are also reported.

Metabolic data have been finally stratified according to patients' TRG, and obtained results are reported in Figure 4. For both kynurenine and serotonin pathways, no statistical differences have been observed, probably due to the low sample size (*n* = 6 and *n* = 7 for TRG 1–2 and TRG 3–5, respectively). Even if a trend of decrease in 5-HT and HIAA tissue levels of TRG 3–5 patients can be inferred from the data, this trend is not supported by gene expression data (Table 4).

## Conclusion

In this work, metabolic and genetic markers of the TRP catabolism before pCRT in LARC patients have been investigated in plasma and tissue samples. In plasma, changes in TRP levels firstly evidenced the difference between responsive (TRG 1–2) and nonresponsive (TRG 3–5) patients. Moreover, TRG 3–5 patients revealed an increased activity along the kynurenine pathway, which correlates with TDO2 overexpression. However, discordant results were obtained from the analysis of the serotonin pathway. Indeed, the decrease in THP1 activity calculated both in plasma and in tissues showed opposite results with respect to the tissue expression. This probably suggests the presence of a posttranscriptional regulation in THP1 protein abundance, which in turn affects its activity in TRG 3–5 patients. Of note, the THP1 posttranscriptional regulation and the diurnal variation of TPH1 activity in the central nervous system have been demonstrated (43–45), but none seems to be reported about cancer. Collectively, these results indicate that mechanisms regulating TRP catabolism may be different between responsive and nonresponsive LARC patients. To confirm these data, further analyses should be performed to increase the sample size and to better investigate the mechanisms involved in tumor response to therapy.

## Data Availability Statement

The original contributions presented in the study are included in the article/Supplementary Material, further inquiries can be directed to the corresponding author/s.

## Ethics Statement

The studies involving human participants were reviewed and approved by Comitato Etico del Centro Oncologico Regionale, Approved Protocol Number: P448. The patients/participants provided their written informed consent to participate in this study.

## Author Contributions

SC: conceptualization and writing - original draft. AF: investigation. CB and AB: investigation and writing - review and editing. VM: writing - review and editing. SP: clinical data management. MA: project supervision. All authors contributed to the article and approved the submitted version.

## Conflict of Interest

The authors declare that the research was conducted in the absence of any commercial or financial relationships that could be construed as a potential conflict of interest.
