# Supplementary material for: Tryptophan Catabolism and Response to Therapy in Locally Advanced Rectal Cancer (LARC) Patients
Source: Front Oncol. 2020 Oct 15;10:583228. doi: 10.3389/fonc.2020.583228 (PMC7593679; doi:10.3389/fonc.2020.583228)
Supplement: Supplementary file 1 [file Data_Sheet_1.docx]

**Supplementary material S1**

**LC-MS/MS analysis:**

List of analytical standards, scheduled transitions and instrumental parameters used in this study. ESI source parameters were as follows: source temperature, 450° C; capillary voltage: 5500 V; curtain gas (nitrogen), 30 psi; nebulizer gas (air) GS1 and GS2, 20 and 30 psi, respectively. DP: declustering potential, CE: collision energy.

| **Analyte** | **Internal Standard** | **Precursor ion (*m/z*)** | **Quantifier ion (*m/z*)** | **Qualifier ion (*m/z*)** | **DP (*V*)** | **CE (*V*)** |
| --- | --- | --- | --- | --- | --- | --- |
| TRPd | - | 210.2 | 150.2 | 192.1 | 23 | 25 |
| TRP | TRPd | 205.2 | 146.2 | 188.2 | 26 | 24 |
| Trypt | TRPd | 161.1 | 144.2 | 117.1 | 30 | 15 |
| 5-HTd | - | 181.1 | 164.2 | 136.3 | 25 | 20 |
| 5-HTP | 5HTd | 211.3 | 162.3 | 134.2 | 25 | 34 |
| 5-HT | 5HTd | 177.1 | 160.2 | 132 | 25 | 30 |
| 5-HIAA | 5HTd | 192 | 146 | 119.2 | 28 | 20 |
| KINAd | - | 195.1 | 149.1 | 167.2 | 25 | 28 |
| KYN | KINAd | 209 | 146 | 174.2 | 23 | 25 |
| KYNA | KINAd | 190.1 | 144.1 | 162.2 | 20 | 28 |
| QA | KINAd | 174 | 128.1 | 146.1 | 22 | 30 |
| XA | KINAd | 206 | 160.2 | 178.2 | 22 | 27 |
| NAm^13^C | - | 129 | 85.1 | 83.4 | 20 | 29 |
| 3-HAA | NAm^13^C | 154 | 136.2 | 80.1 | 32 | 16 |
| NA | NAm^13^C | 124 | 80 | 78 | 20 | 28 |
| NAm | NAm^13^C | 123 | 80 | 78 | 30 | 30 |
| QuiA | NAm^13^C | 168.3 | 150.1 | 124.1 | 25 | 15 |

**Chromatographic conditions:**

Analytes were separated after injection onto an Acclaim 120TM C18 column (2.1x150 mm, 3 μm, ThermoFisher) using a combination of water + 0.05 % TFA (mobile phase A) and acetonitrile + 0.05 % TFA (mobile phase B) at 0.3 mL/min. The chromatographic gradient started with 10 % B (1 min), followed by a linear gradient from 10% to 45% phase B in 5 mins, then 90 % for 1.5 min before column reconditioning. Total gradient run time was 10 mins.

**Supplementary material S2**

**RNA Isolation and quantitative real-time PCR:**

cDNA was synthesized from 1 µg of total RNA using the High-Capacity cDNA Reverse Transcription Kit (Applied Biosystems, Foster City, CA, USA), according to the manufacturer’s protocol by the Veriti^TM^ 96-well Thermal Cycler instrument. qPCR was performed using the 7500 Fast Real-Time PCR System (Applied Biosystems) with Hypoxanthine Phosphoribosyltransferase 1 (HPRT1) gene as endogenous control (Sorby *et al.*, 2010).

The amplification reaction was conducted in a final volume of 10 µl using 50 ng of cDNA, TaqMan® Universal PCR Master Mix 1X (Applied Biosystems) and specific TaqMan® Gene Expression Assay 1X (Applied Biosystems): Hs00984148_m1 (amplicon size: 66 bp) for *IDO1,* Hs00194611_m1 (amplicon size: 74 bp) for *TDO*, Hs00188220_m1 (amplicon size: 94 bp) for *TPH1* and Hs02800695_m1 (amplicon size: 82 bp) for *HPRT1*.

The thermal condition included one cycle at 50° C for 2 min for the UNG incubation and at 95° C for 10 min for the polymerase activation, followed by 40 cycles at 95° C for 15 s for denaturation and at 60° C for 1 min for annealing and extension. Each sample was run in duplicate and the threshold cycle (Ct) average was used for the calculations. The results from each sample were compared against HCT-15 cell line cDNA (as calibrator) using the 2^-ΔΔCt^ calculation method. The fold change was expressed as Relative Quantification (RQ).

**Supplementary material S3**

Calculated instrumental LLOQ, LOD, accuracy %, and CV % for the metabolites under study. For Tryp and 3-HAA, low accuracy (< 85 %) and high CV % (> 20 %) were not satisfying.

| **Analyte** | **LOQ (pg/μl)** | **LOD (pg/μl)** | **Accuracy %** | **CV %** |
| --- | --- | --- | --- | --- |
| TRP | 0.2 | 0.8 | 97 | 10.4 |
| Trypt | 0.5 | 1.6 | 79 | 25.1 |
| 5-HTP | 0.4 | 1.2 | 92 | 4.9 |
| 5-HT | 0.3 | 0.9 | 97 | 8.3 |
| 5-HIAA | 1.4 | 4.6 | 96 | 9.5 |
| KYN | 1.3 | 4.2 | 96 | 13.7 |
| KYNA | 0.9 | 3.1 | 99 | 5.2 |
| QA | 2.6 | 8.6 | 98 | 10.8 |
| XA | 1.9 | 6.5 | 98 | 15.8 |
| 3-HAA | 12.3 | 41.1 | 71 | 25.3 |
| NA | 7.6 | 25.5 | 98 | 3.8 |
| NAm | 10.1 | 33.6 | 98 | 9 |
| QuiA | 13.3 | 44.4 | 98.5 | 18.7 |
